# Supplementary material for: Effect of spironolactone on survival in patients undergoing maintenance hemodialysis
Source: PLoS One. 2024 Mar 29;19(3):e0301458. doi: 10.1371/journal.pone.0301458 (PMC10980200; doi:10.1371/journal.pone.0301458)
Supplement: S1 Table — (DOC) [file pone.0301458.s001.doc]

**S1 Table.** Medication types and Health Insurance Review and Assessment Service codes

| **Medications** | **Codes** |
| --- | --- |
| **Acepril** | 104201ATB, 104202ATB |
| **Amlodipine** | 495901ATB, 459802ACH, 483201ATB, 486501ATB, 107601ATB, 107601ATD, 459801ACH, 459801ATB, 459901ATB, 464601ATB, 470801ATB, 476201ATB, 479701ATB, 483202ATB, 486502ATB, 107602ATB, 107602ATD, 470802ATB |
| **Amlodipine+Atorvastatin** | 614500ATB, 472300ATB472400ATB472500ATB518900ATB |
| **Amlodipine+Losartan+Chlorthalidone** | 662800ATB, 662900ATB, 663000ATB |
| **Amlodipine+Losartan+Rosuvastatin** | 663900ATB, 664000ATB, 664100ATB, 664200ATB, 664300ATB, 664400ATB |
| **Amlodipine+Olmesartan+Rosuvastatin** | 677300ATB, 677400ATB, 677500ATB, 677600ATB |
| **Amlodipine+Rosuvastatin** | 673900ATB, 674000ATB, 674100ATB |
| **Amlodipine+Rosuvastatin+Telmisartan** | 671200ATB, 671300ATB, 671400ATB, 671500ATB, 677000ATB, 677100ATB, 671600ATB, 671700ATB |
| **Amlodipine+Telmisartan+Hydrochlorothiazide** | 663500ATB, 663600ATB, 663700ATB, 663800ATB |
| **Amosulalol** | 107901ATB, 107902ATB |
| **Arotinolol** | 110202ATB, 110201ATB |
| **Atenolol** | 483102ATB, 111402ATB, 483101ATB, 111403ATB |
| **Atenolol+Chlorthalidone** | 262100ATB |
| **Azilsartan** | 662401ATB, 662403ATB, 662402ATB |
| **Azilsartan+Chlorthalidone** | 673500ATB, 673600ATB |
| **Barnidipine** | 114003ACH, 114001ACH, 114002ACH |
| **Benidipine** | 115101ATB, 115102ATB, 115104ATB, 115103ATB |
| **Betaxolol** | 116801ATB, 116803ATB |
| **Bevantolol** | 117002ATB, 117001ATB |
| **Bisoprolol** | 117904ATB, 117903ATB, 117902ATB, 117901ATB |
| **Bisoprolol+Hydrochlorothiazide** | 469800ATB, 470000ATB, 469900ATB |
| **Candesartan** | 122601ATB, 122602ATB, 122603ATB |
| **Candesartan+Amlodipine** | 652900ATB, 653000ATB, 653100ATB |
| **Candesartan+Hydrochlorothiazide** | 423700ATB |
| **Candesartan+Rosuvastatin** | 661800ATB, 661900ATB, 673700ATB, 662000ATB, 662100ATB |
| **Captopril** | 122901ATB, 122902ATB, 122903ATB |
| **Captopril+Hydrochlorothiazide** | 262200ATB, 262300ATB |
| **Carteolol** | 124801ATB |
| **Carvedilol** | 125005ATB, 125003ATB, 662201ATB, 125008ACR, 125001ATB, 662202ATB, 125007ACR, 125002ATB, 125006ACR, 125004ACR |
| **Celiprolol** | 129101ATB |
| **Cilazapril** | 133001ATB, 133002ATB, 133003ATB |
| **Cilnidipine** | 133102ATB, 133101ATB |
| **Clonidine** | 136505ATR |
| **Diltiazem** | 145706ATB, 145707ACR, 145707ATR, 145703ACR, 145706ATR, 145707ATB |
| **Doxazocin** | 149101ATB, 149102ATB, 149104ATR, 149103ATB |
| **Efonidipine** | 441202ATB, 441201ATB |
| **Enalapril** | 151603ATB, 151601ATB |
| **Enalapril+Hydrochlorothiazide** | 453700ATB, 440300ATB |
| **Eprosartan** | 429201ATB |
| **Eprosartan+Hydrochlorothiazide** | 460500ATB |
| **Felodipine** | 157503ATR, 157501ATR |
| **Felodipine+Metoprolol** | 262400ATR |
| **Fimasartan** | 515203ATB, 515201ATB, 515202ATB |
| **Fimasartan+Amlodipine** | 651900ATB, 652000ATB, 652700ATB, 652100ATB |
| **Fimasartan+Hydrochlorothiazide** | 522000ATB, 526800ATB |
| **Fimasartan+Rosuvastatin** | 655000ATB, 654900ATB, 654800ATB, 654700ATB, 654600ATB |
| **Fosinopril** | 163501ATB, 163502ATB |
| **Hydralazine** | 170701ATB |
| **Imidapril** | 173402ATB, 173401ATB |
| **Irbesartan** | 177301ATB, 177303ATB |
| **Irbesartan+Atorvastatin** | 524000ATB, 524100ATB, 527100ATB, 527000ATB |
| **Irbesartan+Hydrochlorothiazide** | 385700ATB, 385800ATB, 553800ATB |
| **Lacidipine** | 180301ATB, 180302ATB, 180303ATB |
| **Lercanidipine** | 182001ATB, 182002ATB |
| **Lisinopril** | 184501ATB |
| **Lisinopril+Hydrochlorothiazide** | 499200ATB, 499300ATB |
| **Losartan** | 185701ATB, 185702ATB |
| **Losartan+Amlodipine** | 503000ATB, 637400ATB, 513900ATB, 637500ATB, 502700ATB, 637600ATB |
| **Losartan+Hydrochlorothiazide** | 262500ATB, 486900ATB, 378900ATB |
| **Manidipine** | 188001ATB, 188002ATB |
| **Metoprolol** | 194003ATR, 193802ATB |
| **Metoprolol+Hydrochlorothiazide** | 262600ATB |
| **Minoxidil** | 196102ATB |
| **Nadolol** | 198301ATB |
| **Nicardipine** | 201003ACR, 201002ATB |
| **Nifedipine** | 201407ACS, 201405ATR, 528201ATR, 201409ATR, 528202ATR, 201401ACS, 201401ATB, 201408ATR |
| **Nimodipine** | 201901ATB, 356202ATR, 356203ATR, 356201ATB, 356202ATB |
| **Nisoldipine** | 356202ATR |
| **Olmesartan** | 468502ATB, 468501ATB, 468503ATB, 520902ATB, 520901ATB |
| **Olmesartan+Amlodipine** | 547800ATB, 632800ATB, 500500ATB, 547700ATB, 629500ATB, 631300ATB, 500600ATB, 547900ATB, 632900ATB, 547600ATB, 548000ATB, 582200ATB, 629600ATB, 633000ATB, 547500ATB, 582400ATB, 629400ATB |
| **Olmesartan+Amlodipine+Hydrochlorothiazide** | 519800ATB, 519700ATB, 520100ATB, 520000ATB, 519900ATB |
| **Olmesartan+Hydrochlorothiazide** | 513600ATB, 489100ATB |
| **Olmesartan+Rosuvastatin** | 644200ATB, 644100ATB, 526900ATB, 526300ATB, 526400ATB, 653200ATB, 526500ATB |
| **Perindopril** | 211301ATB, 501601ATB, 211302ATB, 501602ATB |
| **Perindopril+Indapamide** | 556200ATB |
| **Propranolol** | 219901ATB, 219904ATB, 219906ACR, 219905ACR |
| **Quinapril** | 221901ATB |
| **Ramipril** | 222401ATB, 222402ATB, 222404ATB |
| **Ramipril+Felodipine** | 447100ATB, 447200ATB |
| **Ramipril+Hydrochlorothiazide** | 448600ATB, 448700ATB |
| **Telmisartan** | 378801ATB, 378802ATB, 378803ATB |
| **Telmisartan+Amlodipine** | 521200ATB, 511600ATB, 521300ATB, 511700ATB, 521400ATB, 511500ATB, 644800ATB, 623100ATB |
| **Telmisartan+Hydrochlorothiazide** | 443200ATB, 443300ATB, 502600ATB |
| **Telmisartan+Rosuvastatin** | 631600ATB, 629900ATB, 630000ATB, 631700ATB, 630100ATB, 630200ATB |
| **Temocapril** | 235002ATB |
| **Terazosin** | 235501ATB, 235502ATB, 235503ATB, 616501ATB |
| **Valsartan** | 247103ATB, 247101ATB, 247102ATB, 247104ATB |
| **Valsartan+Amlodipine** | 522600ATB, 492900ATB, 522900ATB, 523200ATB, 522700ATB, 492800ATB, 522800ATB, 523000ATB, 523300ATB, 495800ATB, 523100ATB, 523400ATB |
| **Valsartan+Hydrochlorothiazide** | 356400ATB, 442600ATB |
| **Valsartan+Lercanidipine** | 522200ATB, 522300ATB, 522400ATB |
| **Valsartan+Pitavastatin** | 635000ATB, 635200ATB, 634900ATB, 635100ATB |
| **Valsartan+Rosuvastatin** | 629700ATB, 525000ATB, 525200ATB, 629800ATB, 525100ATB, 525300ATB |
| **Valsartan+Sacubitril** | 651401ATB, 651402ATB, 651403ATB |
| **Verapamil** | 247606ATB, 247607ATB, 247603ATR, 247605ATR, 247601ACR |
| **Zofenopril** | 510401ATB, 510402ATB, 510403ATB |
| **Atorvastatin** | 111501ATB, 111502ATB, 111503ATB, 111504ATB, 502201ATB, 502202ATB, 502203ATB, 502204ATB |
| **Atorvastatin+Amlodipine** | 472300ATB, 472400ATB |
| **Atorvastatin+Ezetimibe** | 633800ATB, 633900ATB, 634800ATB |
| **Fluvastatin** | 162401ACH, 162402ACH, 162403ATR |
| **Lovastatin** | 185801ATB |
| **Pitavastatin** | 470901ATB, 470902ATB, 470903ATB |
| **Pitavastatin+Fenofibrate** | 679300ACH |
| **Pravastatin** | 216601ATB, 216602ATB, 216603ATB, 216604ATB |
| **Rosuvastatin** | 454001ATB, 454002ATD, 454002ATB, 454003ATB, 454003ATD, 454005ATB |
| **Rosuvastatin+Ezetimibe** | 640700ATB, 640800ATB, 640900ATB |
| **Rosuvastatin+Ezetimibe+Telmisartan** | 671400ATB, 671500ATB, 671700ATB |
| **Simvastatin** | 227801ATB, 227802ATB, 227803ATB, 227805ATB, 227806ATB |
| **Aspirin** | 110701ATB, 110702ATB, 110801ATB, 110802ATB, 110902BIJ, 111001ACE, 111001ATB, 111001ATE, 111002ATE, 111003ACE, 111003ATE |
| **Spironolactone** | 262700ATB, 231101ATB, 231102ATB |
| **Polystyrene sulfonate calcium** | 215401APD, 215402ACM, 215402AGN, 215402APD, 215430ASS, 215432ASS, 215402ASS, 215402ASY, 215431CSS |
