# Supplementary material for: Effect of spironolactone on survival in patients undergoing maintenance hemodialysis
Source: PLoS One. 2024 Mar 29;19(3):e0301458. doi: 10.1371/journal.pone.0301458 (PMC10980200; doi:10.1371/journal.pone.0301458)
Supplement: Attachment — Submitted filename: Responses for reviewer comments-complete.docx [file pone.0301458.s002.docx]

Reviewer #1:

1) Is it possible to analyze the determinants of spironolactone prescription?
**Answer: Thank you for your comment. We have performed a logistic regression using the prescription of spironolactone as a dependent variable (Table 2). The added Table 2 is as follows:**

**Table 2. Logistic regression analyses for the use of spironolactone**

|  | **Univariate** | | **Multivariate** | |
| --- | --- | --- | --- | --- |
|  | **OR (SE)** | ***P*** | **OR (SE)** | ***P*** |
| Age (years) | 0.011 (0.004) | 0.013 | -0.022 (0.007) | 0.014 |
| HD vintage (days) | -0.000 (0.000) | <0.001 | -0.000 (0.000) | <0.001 |
| CCI score | 0.163 (0.018) | <0.001 | 0.072 (0.029) | 0.011 |
| Ultrafiltration volume (L/session) | -0.305 (0.050) | <0.001 | -0.175 (0.076) | 0.021 |
| Hemoglobin (g/dL) | -0.146 (0.072) | 0.042 | -0.074 (0.090) | 0.411 |
| Serum albumin (g/dL) | -1.017 (0.153) | <0.001 | -0.895 (0.203) | <0.001 |
| Serum phosphorus (mg/dL) | -0.214 (0.045) | <0.001 | -0.040 (0.064) | 0.534 |
| Serum calcium (mg/dL) | -0.164 (0.063) | 0.009 | 0.043 (0.098) | 0.659 |
| Systolic blood pressure (mmHg) | -0.018 (0.004) | <0.001 | -0.022 (0.006) | <0.001 |
| Diastolic blood pressure (mmHg) | -0.030 (0.007) | <0.001 | -0.002 (0.009) | 0.848 |
| Serum creatinine (mg/dL) | -0.251 (0.022) | <0.001 | -0.186 (0.035) | <0.001 |
| Use of antihypertensive drug | 0.650 (0.143) | <0.001 | 0.196 (0.235) | 0.402 |
| Use of β-blockers | 0.742 (0.114) | <0.001 | 0.737 (0.184) | <0.001 |
| Use of statin | 0.674 (0.114) | <0.001 | 0.490 (0.149) | 0.001 |
| The presence of angina | 0.578 (0.114) | <0.001 | 0.032 (0.161) | 0.841 |
| The presence of MI | 0.774 (0.155) | <0.001 | 0.574 (0.200) | 0.004 |
| The presence of HF | 0.753 (0.115) | <0.001 | 0.469 (0.163) | 0.004 |

*P*-values are tested using Student’s t-test for continuous variables and Pearson’s χ^2^ test for categorical variables. Multivariate analysis is adjusted for age, HD vintage, ultrafiltration volume, hemoglobin, serum albumin, serum phosphorus, serum calcium, systolic blood pressure, diastolic blood pressure, serum creatinine, use of antihypertensive drug, β-blockers, or statin, the presence of angina, MI, or HF.

Control group, patients without spironolactone use; SPR group, patients with spironolactone use.

**Abbreviations:** CCI, Charlson comorbidity index; HD, hemodialysis HF, heart failure; MI, myocardial infarction; OR, odds ratio; SE, standard error.

**Multivariate logistic regression analysis showed that age, HD vintage, ultrafiltration volume, serum albumin, systolic blood pressure, and serum creatinine were inversely associated with the use of spironolactone. The CCI score, the use of β-blocker or statin, or the presence of MI of HF were positively associated with the use of spironolactone. These revealed that patients with underlying heart diseases were prone to taking spironolactone. We have included these variables as covariates in multivariate analyses for mortality. We have added these comments and a Table in the Results section.**

2) Is it possible to mention the differences in patient background from previous studies investigating the impact of spironolactone on patients with HD?

**Answer: Thank you for your comment. Most studies for association between the use of spironolactone and outcomes were performed as randomized trials and most baseline characteristics were similar between the users and non-users. To identify differences in baseline characteristics between the users and non-users, retrospective cross-sectional or observational studies would be more useful than randomized controlled trials. Lin et al. analyzed the nationwide population-based study and evaluated the use of spironolactone and clinical outcomes in patients with advanced chronic kidney disease. In their study, users had a higher the prevalence of heart diseases, CCI scores, the use of statins or aspirin, and erythropoietin doses than non-users [1]. The results of our study showed similar trends to those of Lin’s study. In our study, the use of spironolactone was inversely associated with age, HD vintage, ultrafiltration volume, serum albumin, systolic blood pressure, and serum creatinine, and positively associated with CCI scores, the use of β-blocker or statin, or the presence of MI of HF. Ours and Lin’s studies reveal that patients with underlying heart diseases were prone to taking spironolactone and factors associated with malnutrition or volume overload were also associated with the use of spironolactone. However, due to limitations in sample size of the SPR group, caution is needed in drawing definitive information.**

**Added reference**

**[1] Tseng WC, Liu JS, Hung SC, Kuo KL, Chen YH, Tarng DC, Hsu CC. Effect of spironolactone on the risks of mortality and hospitalization for heart failure in pre-dialysis advanced chronic kidney disease: A nationwide population-based study. Int J Cardiol. 2017 Jul 1;238:72-78.**

3) In line 195, "This indicates that many clinicians hesitate about prescribing spironolactone in patients undergoing HD although it has some beneficial effects."
The benefit of spironolactone administration in patients with HD has been shown in small RCTs, but there is not sufficient evidence. Therefore, I believe that many physicians are not hesitant to prescribe spironolactone, but rather think that 'there is no need to prescribe it.

**Answer: Thank you for your comment. As the reviewer suggested, we have revised the relevant sentence as follows:**

**Recent meta-analyses and some randomized controlled trials showed favorable results of spironolactone on clinical outcomes. However, most randomized studies had small sample sizes and some studies showed a high risk of complications for gynecomastia, hyperkalemia, or arrhythmia by spironolactone. Consequently, there was insufficient evidence regarding the clinical efficacy of spironolactone on cardiovascular outcomes or mortality beyond the hazard effect. Therefore, many clinicians may consider the use of spironolactone unnecessary.**

Reviewer #2: In the present work the authors present data on the impact of MRA intake on survival in patients undergoing chronic hemodialysis (HD). Even though being associated with higher mortality in a univariate analysis, the effect did not remain significant in the multivariate regression. Please find attached my comments.

1) Please provide a Table showing results of both the uni- and multivariate Cox regression model. This important to understand which parameters eliminated MRAs in the multivariate regression.

**Answer: Thank you for your comment. We have added Table 3 with data for covariate and univariate and multivariate analyses. Table 3 is as follows:**

**Table 3.** **Cox regression analyses for patient survival.**

|  | **Univariate** | | **Multivariate** | |
| --- | --- | --- | --- | --- |
|  | **HR (95% CI)** | ***P*** | **HR (95% CI)** | ***P*** |
| Group (ref: control) | 1.34 (1.14–1.58) | <0.001 | 1.06 (0.87–1.30) | 0.588 |
| Age (increase per 1 year) | 1.06 (1.06–1.06) | <0.001 | 1.06 (1.06–1.06) | <0.001 |
| Hemodialysis vintage (increase per 1 day) | 1.00 (1.00–1.01) | <0.001 | 1.00 (1.00–1.01) | <0.001 |
| CCI score (increase per 1 score) | 1.14 (1.13–1.14) | <0.001 | 1.07 (1.07–1.07) | <0.001 |
| Ultrafiltration volume (increase per 1 kg/session) | 0.92 (0.90–0.93) | <0.001 | 1.09 (1.07–1.11) | <0.001 |
| KtV_urea_ (increase per 1 unit) | 0.91 (0.87–0.97) | <0.001 | 0.64 (0.60–0.68) | <0.001 |
| Hemoglobin (increase per 1 g/dL) | 0.86 (0.85–0.88) | <0.001 | 0.92 (0.90–0.94) | <0.001 |
| Serum albumin (increase per 1 g/dL) | 0.37 (0.36–0.39) | <0.001 | 0.63 (0.60–0.66) | <0.001 |
| Serum creatinine (increase per 1 mg/dL) | 0.87 (0.86–0.87) | <0.001 | 0.95 (0.94–0.95) | <0.001 |
| Serum phosphorus (increase per 1 mg/dL) | 0.85 (0.84–0.86) | <0.001 | 1.02 (1.01–1.04) | 0.001 |
| Serum calcium (increase per 1 mg/dL) | 0.94 (0.92–0.95) | <0.001 | 1.05 (1.02–1.07) | <0.001 |
| Systolic blood pressure (increase per 1 mmHg) | 1.01 (1.01–1.01) | <0.001 | 1.01 (1.01–1.01) | <0.001 |
| Diastolic blood pressure (increase per 1 mmHg) | 0.98 (0.98–0.99) | <0.001 | 1.00 (0.99–1.00) | 0.347 |
| Use of antihypertensive drug | 1.12 (1.09–1.15) | <0.001 | 0.94 (0.90–0.98) | 0.006 |
| Use of β-blockers | 1.07 (1.04–1.10) | <0.001 | 1.07 (1.03–1.12) | <0.001 |
| Use of statin | 1.10 (1.07–1.14) | <0.001 | 0.93 (0.90–0.97) | <0.001 |
| Presence of angina | 1.57 (1.53–1.62) | <0.001 | 1.09 (1.05–1.13) | <0.001 |
| Presence of myocardial infarction | 1.90 (1.82–1.98) | <0.001 | 1.20 (1.14–1.27) | <0.001 |
| Presence of heart failure | 1.43 (1.39–1.47) | <0.001 | 1.01 (0.97–1.05) | 0.576 |

Multivariate analysis is adjusted for age, hemodialysis vintage, CCI score, ultrafiltration volume, Kt/V_urea_, hemoglobin, serum albumin, serum creatinine, serum phosphorus, serum calcium, systolic blood pressure, diastolic blood pressure, use of antihypertensive drug, β-blockers, or statin, the presence of angina of myocardial infarction or heart failure, and was performed using enter mode.

**Abbreviations**: CCI, Charlson comorbidity index; CI, confidence interval; HR, hazard ratio.

2) Can the authors provide information regarding the cause of death? Especially cardiovascular death would be of high interest.

**Answer: Thank you for your comment. As the reviewer pointed out, the benefits of spironolactone mainly originate from the cardiovascular system. In our study, the outcome was all-cause mortality, and analyses using death by cardiovascular disease might show different results. Furthermore, information, such as ejection fraction, cardiac mass, or the presence of hypertrophy using echocardiography are very useful to identify the cause-relationship between the use of spironolactone and clinical outcomes beyond simple death. Moreover, left ventricular ejection fraction is very important information for classifying heart failure. Heart failure can be divided into reduced, mildly reduced, or preserved ejection fractions. The effect of spironolactone may be different according to the three groups. However, our study collected from data the HD quality assessment program and claims. HD quality assessment program only collected essential data directly regarding HD quality, such as dialysis adequacy, hemoglobin, or phosphorus levels regardless of the patient status or risk factors associated with prognosis. In addition, the death data was collected using claims data, which included data for survivor or death, but not for cause of death. These are inherent limitations of our study, but our study as a pilot study may be valuable in providing preliminary information to design future studies. We have added these comments in the Discussion section.**

3) Can the authors provide information on the LVEF of each group?

**Answer: Thank you for your comment. We have added some comments for this issue. Detailed explanations are presented in the answer to the previous comment.**

4) Did the patients receive other heart failure medication besides MRAs?

**Answer: Thank you for your comment. We have added the data for β-blockers and renin-angiotensin system blockers (RASB). Patients with β-blockers and RASB were 21370 (39.1%) and 16714 (30.6%) in the control group and 181 (57.4%) and 97 (30.8%) in the SPR group, respectively. The proportion of patients using β-blockers was higher in the SPR group than in the control group (*P* < 0.001). There was no significant difference in RASB use between the two groups (*P* = 0.995). We have added these data in the Results section.**

5) The Kaplan Meier Chart is visually not appealing. Maybe the authors could improve the design of this figure?

**Answer: Thank you for your comment. We have revised Figure 1 to a new figure with a 95% confidence interval, *P*-value, and numbers at risk as follows;**

**Figure 1. Kaplan–Meier curves of patient survival in the two study groups.** SRP: patients with a prescription of spironolactone.

6) Please provide information on follow-up completeness

**Answer: Thank you for your comment. In our study, data during follow-up were collected using claims data and all patients were completely followed up to the end-point. If the patient followed up did not die by the index date (April 2022), they were defined as survivors. If the patient died before the index date, they were defined as non-survivors at the time of death. If the patients performed peritoneal dialysis or kidney transplantation before the index date, we defined the date of the first prescription for peritoneal dialysis or kidney transplantation to the end-point of follow-up and were considered survivors to the date. In addition, we have added numbers at risk in Figure 1 and the data would be useful to identify follow-up status. We have added these data in the Methods section.**

7) Was hyperkalemia more prevalent in the MRA cohort?

**Answer: Thank you for your comment. Our dataset did not include the prevalence of hyperkalemia or serum potassium levels. Hyperkalemia can be developed by various factors such as diet, nutritional status, or the use of many medications. In addition, the ICD-10 code was not added despite hyperkalemia. Therefore, our study did not evaluate the effect of spironolactone on hyperkalemia. Nevertheless, we evaluated the dose of polystyrene sulfonate calcium (PSC), which indirectly revealed the risk of hyperkalemia or serum potassium levels. The dose of PSC was not different between the two groups. Generally, the use of spironolactone is associated with hyperkalemia. Non-difference in the use of PSC between the two groups would be caused by some factors. First, the use of PCS did not directly reveal the hyperkalemia or serum potassium level. Serum potassium level or prevalence of hyperkalemia may be higher in the SPR group than in the control despite of same dose of PCS. The actual use of medication did not match the prescribed doses. Second, considering the risk of hyperkalemia in the SPR group, stricter diet control may be performed. Third, the SPR group had greater comorbidities, and these may be poor nutritional status. The SPR group had lower serum albumin and phosphorus levels compared to the control group. We have added these comments in the Discussion section.**

8) How did the authors deal with patients who received MRAs later during follow-up

**Answer: Thank you for your comment. In our study, 1659 and 3% of the control group were without spironolactone during the HD quality assessment program and with spironolactone after the program. We compared the patient survival according to patients without spironolactone during the program and follow-up, those with spironolactone during the program, and those with spironolactone during follow-up without the drug during the program. Multivariate analysis showed that hazard ratio (95% CI) was 1.08 (95% CI, 0.99-1.18; *P* = 0.073) for patients with spironolactone during follow-up without the drug during the program and 1.06 (95% CI, 0.86-1.31, *P* = 0.563) for those with spironolactone during the program compared to patients without spironolactone during program and follow-up. The small proportions of control may not influence the overall trend. We have added these comments in the Results section.**
