# Supplementary material for: Effect of spironolactone on survival in patients undergoing maintenance hemodialysis
Source: PLoS One. 2024 Mar 29;19(3):e0301458. doi: 10.1371/journal.pone.0301458 (PMC10980200; doi:10.1371/journal.pone.0301458)
Supplement: Attachment — Submitted filename: Responses for reviewer comments.docx [file pone.0301458.s003.docx]

Reviewer #1

If CCI (Charlson comorbidity index) is selected as a covariate when performing multivariate analysis, serum creatinine and the presence of HF or MI should not be included. This is because these items are included in the CCI. CCI should be excluded if serum creatinine and HF or MI are more preferred items.
**Answer: Thank you for your comments. As reviewer pointed out, we have excluded serum creatinine, myocardial infarction, and congestive heart failure as covariates in the multivariate analysis. The revised results were similar with those obtained when including these variables. We have revised the results for multivariate analyses.**

The OR of the logistic analysis in Table 2 should not normally be negative. Is this result the slope of the regression equation?

**Answer: Thank you for your comments. In Table 2, the odd ratio (SE) are presented as regression coefficients and standard errors in logistic regression analysis. We have converted these values to more easily interpretable odds ratio with 95% confidence interval. The revised Table 2 is as follows:**

**Table 2. Logistic regression analyses for the use of spironolactone**

|  | **Univariate** | | | **Multivariate** | | |
| --- | --- | --- | --- | --- | --- | --- |
|  | **OR (95% CI)** | ***P*** | | **OR (95% CI)** | ***P*** |  |
| Age (years) | 1.01 (1.00–1.02) | 0.013 | | 0.98 (0.97–0.99) | 0.029 |  |
| HD vintage (days) | 1.00 (1.00–1.01) | <0.001 | 1.00 (1.00–1.01) | | 0.015 |  |
| CCI score | 1.18 (1.14–1.22) | <0.001 | 1.16 (1.09–1.22) | | <0.001 |  |
| Ultrafiltration volume (L/session) | 0.74 (0.67–0.81) | <0.001 | 0.76 (0.66–0.88) | | <0.001 |  |
| Kt/V_urea_ | 0.84 (0.54–1.32) | 0.446 | 0.66 (0.36–1.22) | | 0.187 |  |
| Hemoglobin (g/dL) | 0.86 (0.75–0.99) | 0.042 | 0.88 (0.72–1.06) | | 0.177 |  |
| Serum albumin (g/dL) | 0.36 (0.27–0.49) | <0.001 | 0.39 (0.25–0.59) | | <0.001 |  |
| Serum phosphorus (mg/dL) | 0.81 (0.74–0.88) | <0.001 | 0.91 (0.80–1.03) | | 0.133 |  |
| Serum calcium (mg/dL) | 0.85 (0.75–0.96) | 0.009 | 1.02 (0.82–1.25) | | 0.878 |  |
| Systolic blood pressure (mmHg) | 0.98 (0.97–0.99) | <0.001 | 0.98 (0.96–0.99) | | <0.001 |  |
| Diastolic blood pressure (mmHg) | 0.97 (0.96–0.98) | <0.001 | 1.00 (0.99–1.02) | | 0.663 |  |
| Use of antihypertensive drug | 1.91 (1.45–2.53) | <0.001 | 0.96 (0.58–1.58) | | 0.874 |  |
| Use of β-blockers | 2.10 (1.68–2.63) | <0.001 | 2.20 (1.48–3.28) | | <0.001 |  |
| Use of statin | 1.96 (1.57–2.45) | <0.001 | 1.88 (1.37–2.58) | | <0.001 |  |
| The presence of angina | 1.78 (1.43–2.23) | <0.001 | 1.12 (0.80–1.55) | | 0.517 |  |

Multivariate analysis was adjusted for age, HD vintage, ultrafiltration volume, Kt/V_urea_, hemoglobin, serum albumin, serum phosphorus, serum calcium, systolic blood pressure, diastolic blood pressure, use of antihypertensive drug, β-blockers, or statin.

Control group, patients without spironolactone use; SPR group, patients with spironolactone use.

**Abbreviations:** CCI, Charlson comorbidity index; CI, confidence interval; HD, hemodialysis; OR, odds ratio.
